# Effect of the Interaction between Obstructive Sleep Apnea and Lipoprotein(a) on Insulin Resistance: A Large-Scale Cross-Sectional Study

**DOI:** 10.1155/2019/9583286

**Published:** 2019-04-08

**Authors:** Yupu Liu, Juanjuan Zou, Xinyi Li, Xiaolong Zhao, Jianyin Zou, Suru Liu, Lili Meng, Yingjun Qian, Huajun Xu, Hongliang Yi, Jian Guan, Shankai Yin

**Affiliations:** ^1^Department of Otolaryngology, Therapy Center for Obstructive Sleep Apnea, Shanghai Jiao Tong University Affiliated Sixth People's Hospital, 600 Yishan Road, Xuhui District, Shanghai 200233, China; ^2^Otolaryngology Institute of Shanghai Jiao Tong University, China; ^3^Shanghai Key Laboratory of Sleep Disordered Breathing, China

## Abstract

Both obstructive sleep apnea (OSA) and decreased serum lipoprotein(a) (Lp(a)) concentrations are associated with insulin resistance. However, their interaction effect on insulin resistance has never been investigated. Therefore, we performed a cross-sectional study on OSA-suspected Chinese Han participants. Laboratory-based polysomnographic variables, biochemical indicators, anthropometric measurements, and medical history were collected. Linear regression and binary logistic regression analyses with interaction terms were used to investigate the potential effects of the interaction between the severity of OSA (assessed by the apnea-hypopnea index (AHI)) and Lp(a) concentrations on insulin resistance (assessed by the homeostasis model assessment of insulin resistance (HOMA-IR)), after adjusting for potential confounders including age, gender, body mass index, waist-to-hip circumference ratio, mean arterial pressure, smoking status, drinking status, and lipid profiles. A total of 4,152 participants were enrolled. In the OSA-suspected population, AHI positively correlated with insulin resistance and serum Lp(a) concentrations independently and inversely correlated with insulin resistance. In addition, the interaction analysis showed that the linear association between lgAHI and lgHOMA-IR was much steeper and more significant in subjects with relatively low Lp(a) concentrations, suggesting a significant positive interaction between lgLp(a) and lgAHI on lgHOMA-IR (*P* = 0.013). Furthermore, the interaction on a multiplicative scale also demonstrated a significant positive interaction (*P* = 0.044). A stronger association between AHI quartiles and the presence of insulin resistance (defined as HOMA-IR > 3) could be observed for participants within lower Lp(a) quartiles. In conclusion, a significant positive interaction was observed between OSA and decreased Lp(a) with respect to insulin resistance. This association might be relevant to the assessment of metabolic or cardiovascular disease risk in OSA patients.

## 1. Introduction

Obstructive sleep apnea (OSA) is the most common form of sleep-disordered breathing and is characterized by recurrent episodes of complete or partial upper airway obstruction during sleep [1]. These obstructive events lead to multiple adverse physiological changes, including intermittent hypoxia, sleep fragmentation, inflammation, oxidative stress, and increased sympathetic tone. Together, these changes could predispose OSA patients to a higher risk of insulin resistance [2–5], a pathological condition which plays important roles in the pathogenesis of various metabolic and cardiovascular diseases (CVD), including metabolic syndrome, type 2 diabetes, hypertension, atherosclerosis, and stroke [6–8].

Low serum lipoprotein(a) (Lp(a)) concentration is recognized as another risk factor for insulin resistance [9–14]. Lp(a) is a highly atherogenic lipoprotein composed of a low-density lipoprotein- (LDL-) like particle covalently bound to apo(a) [15]. Its serum concentrations have a more than 1,000-fold interindividual range and are primarily genetically determined by the LPA gene without other major anthropometric, dietary, or environmental influences [15–19]. Recently, observational studies demonstrated that decreased Lp(a) is associated with a higher risk of insulin resistance in several populations including diabetic, nondiabetic, hypertensive, and dyslipidemic patients, indicating that low Lp(a) levels may be associated with the development of insulin resistance [9–14, 20].

However, to our knowledge, no prior studies have investigated whether Lp(a) is related to insulin resistance in individuals with suspected OSA. In addition, it is also unknown whether there is any interaction between Lp(a) and OSA with respect to the severity of insulin resistance. These questions are of potential clinical importance as they are relevant to the assessments of metabolic and cardiovascular disease risk in OSA patients. For instance, because Lp(a) is highly atherogenic, it has been recommended that its concentrations should be lowered in individuals with a high risk of CVD [21–23]. However, this treatment may lead to an excessive risk of insulin resistance and subsequent adverse metabolic or cardiovascular consequences in OSA patients [24–26].

Therefore, we set up a cross-sectional study of 5,479 OSA-suspected participants with the aims to (1) investigate the relationships between Lp(a) and insulin resistance in an OSA-suspected population and (2) test for an interaction effect of Lp(a) and OSA on insulin resistance.

## 2. Methods

### 2.1. Study Population

Participants were enrolled from the Shanghai Sleep Health Study cohort, which included a total of 5,479 unrelated consecutive suspected OSA subjects who were referred to the Sleep Center of the Affiliated Sixth People's Hospital, Shanghai Jiao Tong University, from January 2007 to June 2017. Participants were mainly residents of cities in southern China, and all were Han Chinese. Informed consent was obtained in writing from each participant according to the guidance of the National Ethics Regulation Committee of China. This study was approved by the Institutional Ethics Committee of the Sixth Affiliated Hospital of Shanghai Jiao Tong University.

The exclusion criteria were as follows: (1) age less than 18 years; (2) history of OSA diagnosis or treatment; (3) use of lipid-lowering drugs; (4) use of insulin or oral hypoglycemic agents; (5) systemic steroid treatment or hormone-replacement therapy; (6) severe comorbid diseases, such as congestive heart failure, psychiatric disturbances, chronic liver disease, or chronic kidney disease; (7) acute inflammation; (8) recorded total sleep time < 4 h; (9) sleep disorders other than OSA, such as central sleep apnea or narcolepsy; and (10) unavailable clinical data. Finally, 1,327 met the exclusion criteria and were excluded from the study.

### 2.2. Medical History and Physical Measurements

Before overnight polysomnography (PSG), all participants were asked to complete a uniform questionnaire regarding medical histories, smoking status, and drinking status. Current smoking or drinking was defined as regular consumption of cigarettes or alcohol in the past six months [27]. Height and weight were measured using standard anthropometric methods, with the participants dressed in lightweight clothing and with bare feet. Body mass index (BMI) was calculated as weight divided by height squared (kg/m^2^). Blood pressure was measured with participants in a seated position after a 5 min rest and recorded as the average value of three sequential measurements at 1 min intervals. Systolic blood pressure (SBP) and diastolic blood pressure (DBP) were recorded. Mean arterial pressure (MAP) was calculated as (SBP + 2 ∗ DBP)/3.

### 2.3. Overnight Polysomnography Parameters

Overnight PSG was conducted in the sleep center of the Shanghai Jiao Tong University Affiliated Sixth People's Hospital with an Alice 4 system (Philips Respironics Inc., Pittsburgh, PA, USA). Data recorded included electroencephalography, electrooculography, submental electromyogram, oronasal airflow (nasal pressure and thermistor), chest and abdominal movements, electrocardiogram, peripheral SpO_2_, and body positions. All recordings were analyzed manually according to standard criteria by the same skilled technician [28]. Apnea was defined as the complete cessation of airflow lasting ≥10 s. Hypopnea was defined as either a decrease of airflow ≥ 50% for a duration of ≥10 s or a decrease of airflow < 50% but accompanied by a decrease in SaO_2_ ≥ 4% or an arousal. The apnea-hypopnea index (AHI) was defined as the total number of apneas and hypopneas per hour during sleep. OSA was diagnosed as AHI ≥ 5 events per hour.

### 2.4. Biochemical Indicators

For each participant, a fasting blood sample was drawn from the antecubital vein in the morning after polysomnographic monitoring. Blood samples were assayed by laboratory staff in our hospital unaware of participants' disease status. Serum Lp(a) concentrations were measured using a high-sensitivity immunoturbidimetric assay which was able to detect Lp(a) concentrations from 0 to 100 mg/dL. Blood samples with Lp(a) > 100 mg/dL were routinely diluted 1 : 10. The other biochemical indicators (fasting insulin and fasting blood glucose) and lipid profiles (total cholesterol (TC), triglycerides (TG), high-density lipoprotein cholesterol (HDL-C), low-density lipoprotein cholesterol (LDL-C), apolipoprotein A-I (ApoA-I), and apolipoprotein B (ApoB)) were quantified according to manufacturer specifications using a Hitachi H-7600 analyzer.

The severity of insulin resistance was quantified by calculating the homeostasis model assessment of insulin resistance (HOMA-IR, calculated as the product of fasting glucose (mmol/L) and fasting insulin (mU/L) divided by 22.5). Insulin resistance was defined as HOMA-IR > 3.0 [29]. HOMA-IR correlates well with the hyperinsulinemia euglycemic clamp (the gold standard measure of insulin resistance), thus enabling accurate identification of individuals with insulin resistance [30].

### 2.5. Statistical Analysis

Descriptive variables were expressed as means ± SD, medians (interquartile ranges), or percentages, as appropriate. Baseline characteristics were compared among the Lp(a) quartiles or AHI quartiles using the Kruskal-Wallis *H* test, one-way analysis of variance (ANOVA), or the chi-squared test according to the distribution characteristics of the data. Variables with nonnormal distributions were log-transformed before analysis. The polynomial linear trend test was used to assess linear trends across Lp(a) quartiles or AHI quartiles for continuous variables, and the linear-by-linear association test was used for dichotomous variables. Simple and multiple linear regression analyses were used to investigate the association of AHI, serum Lp(a) concentration, and HOMA-IR. Binary logistic regression analyses were used to determine the odds ratios (ORs) and 95% confidence intervals (95% CIs) of the presence of insulin resistance across Lp(a) quartiles and AHI quartiles, using the top quartile of Lp(a) or the bottom quartile of AHI as the reference. All continuous variables were examined for linearity before logistic regression. Furthermore, the effect of the interaction between Lp(a) and AHI (as continuous variables) on HOMA-IR was assessed with linear regression analysis by adding an interaction term to the regression model. The multiplicative interaction between quartiles of Lp(a) and AHI with respect to insulin resistance was assessed with logistic regression analysis. Statistical analyses were performed using SPSS v.24.0.0 (IBM Corp., Armonk, NY, USA) and R software package 3.4.1 (http://www.r-project.org/). All *P* values given are two-sided, with the significance level set to 0.05.

## 3. Results

### 3.1. Basic Characteristics of Participants

Among the 4,152 included participants, 79.0% were found to have OSA and 21.0% were not, 79.6% were men and 20.4% were women, the median age was 41.0 years, the median Lp(a) concentration was 7.40 mg/dL, and the median AHI was 27.9 events/h. There were 1,595 participants with insulin resistance, and the prevalence rate was 38.4%.

### 3.2. Relationship between AHI and Insulin Resistance in an OSA-Suspected Population

The general characteristics of all participants categorized by AHI quartiles is shown in Table 1. Participants in the lowest to highest quartiles of AHI had a median AHI of 2.2, 16.2, 42.5, and 70.3 events/h, respectively. After multiple adjustments, lgAHI was independently and positively associated with lgHOMA-IR (Table 2; *β* = 0.070, *P* < 0.001). The adjusted odds ratios for insulin resistance across AHI quartiles 2–4 were 1.61, 1.92, and 2.87, respectively, as compared to the lowest AHI quartile (Table 3; *P* for linear trend < 0.001), indicating an independent, positive relationship between OSA severity and insulin resistance.

### 3.3. Relationship between Lp(a) and Insulin Resistance in an OSA-Suspected Population

The general characteristics of participants according to the quartiles of serum Lp(a) concentrations are summarized in Table 4, and the *P* value for each relationship was calculated using each quartile of serum Lp(a) concentrations taken as a unit. Participants in the lowest to highest quartiles of Lp(a) had median serum Lp(a) concentrations of 2.50, 5.50, 10.40, and 25.10 mg/dL, respectively. Across the four quartiles, a significant increasing trend was found regarding age and fasting serum concentrations of TC, LDL-C, HDL-C, and ApoB (Table 4). Meanwhile, a significant decreasing trend was found regarding the percentage of male participants; AHI; BMI; fasting serum concentrations of glucose, insulin, and TG; HOMA-IR; and the percentage of participants with insulin resistance (Table 4). As shown, from the lowest Lp(a) quartile to the highest, a decreasing trend in HOMA-IR (from 2.74 to 2.22, *P* for linear trend < 0.001) and the presence of insulin resistance (44.4% to 33.0%, *P* for linear trend < 0.001) was observed.

Linear regression analysis demonstrated that lgLp(a) significantly and inversely correlated with lgHOMA-IR, both in the unadjusted model (Table 2, *β* = −0.067, *P* < 0.001) and the multivariable-adjusted model incorporating potential confounders including AHI, age, gender, BMI, waist-to-hip circumference ratio, MAP, current smoking status, current drinking status, and lipid profiles (Table 2, *β* = −0.034, *P* < 0.001). In addition, lgLp(a) was also inversely and independently correlated with lgFBG (fasting blood glucose; *β* = −0.007, *P* = 0.004) and lgFSI (fasting serum insulin; *β* = −0.026, *P* < 0.001) after adjustment.

With the highest quartile of Lp(a) as the reference group, univariate logistic regression showed significantly increased odds ratios for the presence of insulin resistance from Lp(a) quartile 4 to quartile 1 (Table 3, *P* for linear trend < 0.001). After further adjustment for confounding factors, the trend remained significant (*P* for linear trend = 0.029), with odds ratios of 1.16 (95% CI: 0.93–1.43) for Lp(a) quartile 3, 1.21 (95% CI: 0.98–1.50) for Lp(a) quartile 2, and 1.40 (95% CI: 1.12–1.74) for Lp(a) quartile 1, suggesting an inverse relationship between the Lp(a) level and insulin resistance in this OSA-suspected population.

### 3.4. Interaction between OSA and Lp(a) with regard to Insulin Resistance

**Figure 1:**
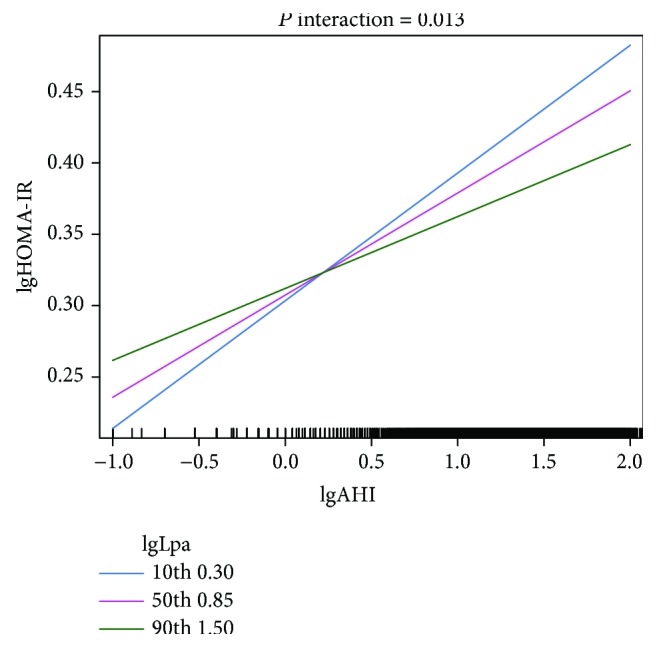
Interaction between lgAHI and lgLp(a) on the relationship with lgHOMA-IR. As depicted, the relationship between lgAHI and lgHOMA-IR is modulated by the level of lgLp(a). With lgLp(a) declining (from the 90th to 10th percentile), a more steep and significant positive linear relationship between lgAHI and lgHOMA-IR could be observed, suggesting significant positive interaction. The *P* value was calculated with multivariable linear regression analyses adjusted for age, gender, BMI, waist-to-hip circumference ratio, MAP, current smoking status, current drinking status, TC, TG, HDL-C, LDL-C, ApoA-I, and ApoB.

As both increased AHI and decreased Lp(a) were correlated with a higher degree of insulin resistance, we next aimed to investigate whether there is a potential interaction effect of these two factors on insulin resistance. As shown in Figure 1, as Lp(a) concentrations decline (from the 90th to the 10th percentile), a steeper and more significant positive linear relationship between lgAHI and lgHOMA-IR is observed, suggesting a significant positive interaction between lgLp(a) and lgAHI on the lgHOMA-IR level (*P* interaction = 0.013, after multivariable adjustment including age, gender, BMI, waist-to-hip circumference ratio, MAP, current smoking status, current drinking status, and lipid profiles).

**Figure 2:**
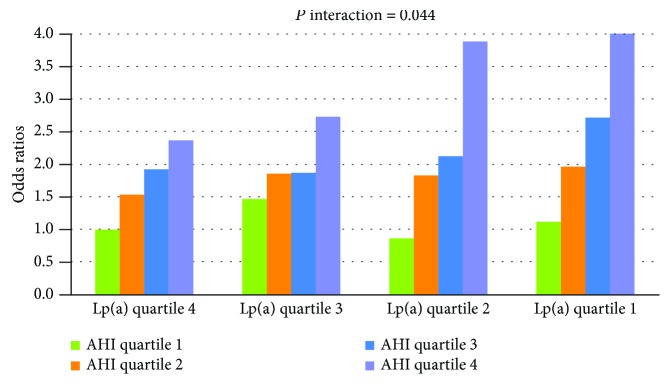
Multiplicative interaction between AHI quartiles and Lp(a) quartiles on the association with the presence of insulin resistance. More significant trends in the relationship between AHI quartiles and insulin resistance were found in the lower Lp(a) quartiles, indicating significant positive interaction. Odds ratios and *P* values were calculated with logistic regression analyses after adjusting for age, gender, BMI, waist-to-hip circumference ratio, MAP, current smoking status, current drinking status, TC, TG, HDL-C, LDL-C, ApoA-I, and ApoB. AHI quartiles 1-4 correspond to the AHI range of ≤7, 7.1-28, 28.1-57, and >57 events/h, respectively. Lp(a) quartiles 1-4 correspond to the serum Lp(a) concentration range of ≤3.90, 3.91-7.40, 7.41-15.10, and >15.10 mg/dL, respectively.

With multivariable-adjusted logistic regression analysis, we also found a significant positive interaction on a multiplicative scale between quartiles of Lp(a) and AHI with respect to the presence of insulin resistance (Figure 2, *P* interaction = 0.044). More significant trends in the relationship between AHI quartiles and the presence of insulin resistance were observed in subjects within lower Lp(a) quartiles (ORs of insulin resistance across AHI quartiles within Lp(a) quartile 4 were 1.00, 1.53, 1.92, and 2.36; those within Lp(a) quartile 3 were 1.47, 1.85, 1.87, and 2.72; those within Lp(a) quartile 2 were 0.86, 1.82, 2.12, and 3.88; and those within Lp(a) quartile 1 were 1.12, 1.96, 2.71, and 4.01).

## 4. Discussion

With this large-scale cross-sectional study, we first found an independent and inverse relationship between serum Lp(a) levels and the severity of insulin resistance as assessed by HOMA-IR in a population with suspected OSA, after adjustment for multiple potential confounders (Tables 2 and 3). Furthermore, the data revealed that as Lp(a) concentrations decline, the relationship between AHI and insulin resistance is steeper and more significant, suggesting a significant positive interaction between Lp(a) and AHI with respect to insulin resistance (Figures 1 and 2).

Lp(a) is a complex lipoprotein composed of an LDL-like particle covalently bound to apo(a) [15]. Its serum concentrations, which have a more than 1,000-fold interindividual range, are primarily genetically determined by the LPA gene and are thought to remain stable throughout the lifetime without other major influences [15–19]. The exact physiological function of Lp(a) is complex and diverse. First, it is demonstrated to be highly atherogenic; epidemiological and genetic evidence strongly indicates that elevated Lp(a) is a strong causal risk factor for CVD [21–23]. However, more recent observational studies have demonstrated that decreased Lp(a) is associated with a higher risk of insulin resistance in diabetic, nondiabetic, hypertensive, and dyslipidemic populations. In line with previous studies [9–14, 20], our results demonstrated that Lp(a) was independently and negatively associated with the degree of insulin resistance in an OSA-suspected population.

The mechanism of this inverse association is still unclear. Kamstrup and Nordestgaard suggested that the large Lp(a) isoform size, which is exhibited in subjects with low Lp(a) levels, may elevate the activity of lipoprotein-associated phospholipase A2 (Lp-PLA2) [20], which stimulates the production of inflammatory cytokines in adipose tissue and leads to insulin resistance [31]. This implies that, in addition to the direct atherogenic role of elevated Lp(a), decreased Lp(a) might in turn contribute to metabolic and cardiovascular risk through insulin resistance [6–8, 26]. However, a “reverse causation” could not be excluded, as several studies have shown that insulin may have a direct effect on serum Lp(a) levels. Haffner et al. found that in patients with type 1 diabetes, insulin therapy was associated with a significant reduction in Lp(a) levels [32]. Additionally, Neele et al. found that a high level of insulin decreased the synthesis of apo(a), a component of Lp(a) [33]. Conversely, a string of studies conducted in patients with type 2 diabetes opposed a direct effect of insulin on Lp(a) levels because no significant decline in Lp(a) was observed after oral hypoglycemic treatment, despite significant improvement in glucometabolism traits [34–36]. In summary, further studies are needed to clarify the underlying pathophysiological mechanism of the inverse relationship between Lp(a) and insulin resistance, as it may point to new targets for the prevention and treatment of insulin resistance and subsequent metabolic and cardiovascular diseases.

Furthermore, in the present study, we observed a significant positive interaction between increased AHI and decreased Lp(a) with respect to the severity of insulin resistance. As Lp(a) concentrations declined, a steeper and more significant positive relationship between AHI and insulin resistance was observed (Figures 1 and 2). This suggests that decreased Lp(a) concentrations, which are primarily genetically determined and remain stable over an individual's lifetime [15, 16], may biologically enhance the adverse pathophysiological effects of OSA on insulin resistance. Our finding is of clinical importance, as it may be relevant to the assessment of metabolic or cardiovascular disease risk in patients with OSA. For example, it has been recommended that Lp(a) concentrations should be lowered in individuals with a high risk of CVD; however, any treatment that lowers Lp(a) levels to reduce CVD risk may lead to an increased risk of insulin resistance and subsequent adverse metabolic and cardiovascular consequences in patients with untreated OSA [24–26]. Therefore, we suggest that to fully evaluate the risk of metabolic and cardiovascular diseases, the effect of the interaction between Lp(a) and OSA on insulin resistance must be appropriately considered. Besides, extra caution should be applied in interpreting our results, because multivariable linear and logistic analyses both showed that the positive relationship between AHI and HOMA-IR was independent of Lp(a) levels. Therefore, interaction between Lp(a) and AHI observed in this study may potentially benefit risk stratification in patients with suspected OSA; however, it should not influence treatment decision-making.

To the best of our knowledge, this is the first study to assess the association of Lp(a) concentrations and insulin resistance in an OSA-suspected population, as well as to explore the interaction of Lp(a) and AHI on the severity of insulin resistance. Our study has several strengths, including the large sample size, standard PSG recording, and fully adjusted models evaluating interaction effects, which increase the quality of our evidence. Meanwhile, some limitations must be stated. First, because of the cross-sectional study design, causality between AHI, Lp(a), and insulin resistance cannot be inferred. Second, Lp(a) kringle, renal function, inflammation, physical activity, daily alcohol consumption dose, and diet are factors shown to influence both Lp(a) levels and insulin resistance [37–40]. Regretfully, our study was not specifically designed to cover these potential confounders, so their effects could not be accounted for in the analysis. Third, cardiovascular damage or outcomes were not measured. Trials incorporating these outcomes are needed in the future to fully depict the joint effect of OSA, Lp(a), and insulin resistance. Finally, possible discrepancy between statistical effects and biological effects might exist. Further fundamental and intervention studies are needed to confirm the biological or clinical impact of our findings.

## 5. Conclusions

In an OSA-suspected population, serum Lp(a) concentrations inversely and independently correlated with insulin resistance following extensive adjustment. In addition, a significant positive interaction was noted between OSA severity and decreased Lp(a) with respect to the degree of insulin resistance. Proper evaluation of the metabolic and cardiovascular disease risk in OSA patients requires appropriate consideration of these associations. Our results also warrant further investigation into the mechanisms underlying the exacerbation of insulin resistance across OSA severities with declining Lp(a).

## Figures and Tables

**Table 1:** Characteristics of participants by AHI quartiles.

Events/h	Quartile 1	Quartile 2	Quartile 3	Quartile 4	*P* for trend
	AHI ≤ 7	7 < AHI ≤ 28	28 < AHI ≤ 57	AHI > 57	
*N*	1,053	1,031	1,017	1,051	—
Age (years)	37.0 (30.0, 47.0)	42.0 (33.0, 53.0)	44.0 (35.0, 55.0)	40.0 (33.0, 49.0)	<0.001
Male (%)	638 (60.6%)	812 (78.8%)	896 (88.1%)	959 (91.2%)	<0.001
BMI (kg/m^2^)	23.8 (21.8, 25.9)	25.7 (23.7, 27.7)	26.6 (24.9, 28.7)	28.6 (26.5, 31.0)	<0.001
Waist-to-hip ratio	0.90 (0.85, 0.94)	0.94 (0.90, 0.97)	0.95 (0.92, 0.99)	0.97 (0.94, 1.00)	<0.001
SBP (mmHg)	120 (111, 125)	120 (114, 130)	125 (118, 135)	126 (120, 137)	<0.001
DBP (mmHg)	78 (70, 85)	80 (70, 85)	80 (74, 87)	80 (77, 90)	<0.001
MAP (mmHg)	91.7 (84.3, 95.0)	93.3 (86.3, 99.3)	94.3 (89.0, 102.3)	96.7 (91.0, 104.7)	<0.001
AHI (events/h)	2.2 (0.8, 4.2)	16.2 (11.0, 21.5)	42.5 (34.8, 49.7)	70.3 (63.7, 79.1)	<0.001
Lp(a) (mg/dL)	7.83 (4.10, 16.80)	7.40 (3.90, 14.26)	7.90 (4.18, 16.20)	6.50 (3.46, 13.10)	<0.001
FBG (mmol/L)	5.03 (4.69, 5.33)	5.16 (4.84, 5.55)	5.31 (4.96, 5.79)	5.48 (5.10, 6.10)	<0.001
FSI (IU/mL)	7.5 (5.2, 6.1)	9.8 (6.6, 14.4)	11.0 (7.4, 16.2)	14.6 (10.1, 20.4)	<0.001
HOMA-IR	1.63 (1.11, 2.48)	2.27 (1.40, 3.38)	2.62 (1.75, 4.10)	3.63 (2.44, 5.35)	<0.001
Insulin resistance (%)	169 (16.0%)	334 (32.4%)	424 (41.7%)	668 (63.6%)	<0.001
TC (mmol/L)	4.35 (3.76, 4.95)	4.60 (4.05, 5.22)	4.77 (4.20, 5.34)	4.85 (4.29, 5.48)	<0.001
TG (mmol/L)	1.13 (0.76, 1.61)	1.50 (1.05, 2.16)	1.62 (1.15, 2.30)	1.83 (1.34, 2.71)	<0.001
LDL-C (mmol/L)	2.66 (2.14, 3.20)	2.92 (2.43, 3.42)	3.00 (2.50, 3.51)	3.12 (2.59, 3.68)	<0.001
HDL-C (mmol/L)	1.10 (0.96, 1.30)	1.04 (0.91, 1.20)	1.02 (0.90, 1.17)	1.00 (0.87, 1.13)	<0.001
ApoA-I (g/L)	1.10 (0.97, 1.24)	1.06 (0.95, 1.21)	1.07 (0.96, 1.20)	1.06 (0.96, 1.19)	<0.001
ApoB (g/L)	0.74 (0.63, 0.86)	0.81 (0.71, 0.94)	0.84 (0.74, 0.96)	0.87 (0.76, 0.99)	<0.001
Current smoker (%)	135 (12.8%)	203 (19.7%)	237 (23.3%)	239 (22.7%)	<0.001
Current drinker (%)	106 (10.1%)	127 (12.3%)	153 (15.0%)	166 (15.8%)	<0.001

BMI: body mass index; SBP: systolic blood pressure; DBP: diastolic blood pressure; MAP: mean arterial pressure; AHI: apnea-hypopnea index; FBG: fast blood glucose; FSI: fast serum insulin; HOMA-IR: homeostasis model assessment of insulin resistance; TC: total cholesterol; TG: total triglyceride; LDL-C: low-density lipoprotein cholesterol; HDL-C: high-density lipoprotein cholesterol. Insulin resistance: HOMA-IR > 3.0.

**Table 2:** The association between lgAHI and lgLp(a) with insulin resistance-related traits.

Dependent	Independent lgAHI				Independent lgLp(a)			
	*B*	SE (*β*)	Beta	*P*	*B*	SE (*β*)	Beta	*P*
*Unadjusted*								
lgHOMA-IR	0.188	0.007	0.402	<0.001	−0.067	0.011	−0.098	<0.001
lgFBG	0.033	0.002	0.276	<0.001	−0.010	0.003	−0.060	<0.001
lgFSI	0.156	0.006	0.371	<0.001	−0.058	0.009	−0.094	<0.001
*Adjusted*								
lgHOMA-IR	0.070	0.007	0.150	<0.001	−0.034	0.009	−0.050	<0.001
lgFBG	0.012	0.002	0.103	<0.001	−0.007	0.003	−0.044	0.004
lgFSI	0.058	0.006	0.139	<0.001	−0.026	0.008	−0.044	<0.001

Values were calculated with unadjusted and multivariable-adjusted linear regression analyses. Adjusted variables include Lp(a) or AHI, age, gender, BMI, waist-to-hip circumference ratio, MAP, current smoking status, current drinking status, TC, TG, HDL-C, LDL-C, ApoA-I, and ApoB.

**Table 3:** The odds ratios for the presence of insulin resistance across AHI or Lp(a) quartiles.

	Quartile 1	Quartile 2	Quartile 3	Quartile 4	*P* for linear trend
*AHI quartiles*					
*n* (cases/participants)	169/1,053	334/1,031	424/1,017	668/1,051	—
Unadjusted	1	2.51 (2.03, 3.10)	3.74 (3.05, 4.61)	9.12 (7.43, 11.25)	<0.001
Adjusted	1	1.61 (1.27, 2.05)	1.92 (1.50, 2.46)	2.87 (2.23, 3.70)	<0.001
*Lp(a) quartiles*					
*n* (cases/participants)	456/1,026	427/1,062	371/1,032	341/1,032	—
Unadjusted	1.62 (1.36, 1.94)	1.35 (1.14, 1.63)	1.14 (0.95, 1.36)	1	<0.001
Adjusted	1.40 (1.12, 1.74)	1.21 (0.98, 1.50)	1.16 (0.93, 1.43)	1	0.029

Odds ratios, 95% confidence intervals, and *P* values were calculated using logistic regression analyses. Insulin resistance was defined as the HOMA-IR index > 3. AHI quartiles 1-4 correspond to the AHI range of ≤7, 7.1-28, 28.1-57, and >57 events/h, respectively. Lp(a) quartiles 1-4 correspond to the serum Lp(a) concentration range of ≤3.90, 3.91-7.40, 7.41-15.10, and >15.10 mg/dL, respectively. Adjusted variables include Lp(a) or AHI quartiles, age, gender, BMI, waist-to-hip circumference ratio, MAP, current smoking status, current drinking status, TC, TG, HDL-C, LDL-C, ApoA-I, and ApoB.

**Table 4:** Characteristics of participants by Lp(a) quartiles.

mg/dL	Quartile 1	Quartile 2	Quartile 3	Quartile 4	*P* for trend
	Lp(a) ≤ 3.90	3.90 < Lp(a) ≤ 7.40	7.41 < Lp(a) ≤ 15.10	Lp(a) > 15.10	
*N*	1,026	1,062	1,032	1,032	—
Age (years)	39.0 (32.0, 49.0)	40.0 (33.0, 51.0)	42.0 (33.0, 53.0)	42.0 (34.0, 53.0)	<0.001
Male (%)	842 (82.1%)	854 (80.4%)	819 (79.4%)	790 (76.6%)	0.002
BMI (kg/m^2^)	26.5 (24.1, 29.4)	26.4 (23.9, 29.1)	26.2 (24.0, 28.4)	25.7 (23.8, 28.4)	<0.001
Waist-to-hip ratio	0.95 (0.90, 0.98)	0.94 (0.90, 0.98)	0.94 (0.90, 0.98)	0.94 (0.90, 0.98)	0.045
SBP (mmHg)	123 (117, 134)	120 (115, 130)	122 (116, 132)	120 (115, 131)	0.061
DBP (mmHg)	80 (73, 86)	80 (72, 85)	80 (73, 86)	80 (71, 85)	0.043
MAP (mmHg)	93.3 (88.0, 101.7)	93.3 (87.7, 99.8)	93.3 (88.0, 101.3)	93.3 (86.7, 100.0)	0.041
AHI (events/h)	31.3 (8.4, 62.2)	28.4 (7.3, 58.6)	26.6 (7.3, 55.9)	26.3 (5.9, 53.1)	<0.001
Lp(a) (mg/dL)	2.50 (1.70, 3.20)	5.50 (4.60, 6.40)	10.40 (8.80, 12.40)	25.10 (19.50, 36.78)	<0.001
FBG (mmol/L)	5.28 (4.90, 5.73)	5.24 (4.90, 5.72)	5.22 (4.88, 5.68)	5.18 (4.87, 5.62)	<0.001
FSI (IU/mL)	11.2 (7.4, 17.2)	10.7 (7.0, 16.1)	10.0 (6.7, 15.3)	9.7 (6.6, 14.6)	<0.001
HOMA-IR	2.74 (1.65, 4.25)	2.53 (1.58, 3.91)	2.34 (1.50, 3.80)	2.22 (1.49, 3.50)	<0.001
Insulin resistance (%)	456 (44.4%)	427 (40.2%)	371 (35.9%)	341 (33.0%)	<0.001
TC (mmol/L)	4.54 (3.98, 5.18)	4.57 (4.00, 5.22)	4.66 (4.08, 5.29)	4.79 (4.22, 5.34)	<0.001
TG (mmol/L)	1.68 (1.09, 2.48)	1.57 (1.05, 2.29)	1.45 (1.04, 2.00)	1.41 (1.01, 2.01)	<0.001
LDL-C (mmol/L)	2.79 (2.27, 3.35)	2.88 (2.36, 3.40)	2.97 (2.47, 3.51)	3.06 (2.59, 3.56)	<0.001
HDL-C (mmol/L)	1.03 (0.89, 1.18)	1.02 (0.90, 1.18)	1.04 (0.91, 1.20)	1.06 (0.93, 1.23)	0.002
ApoA-I (g/L)	1.07 (0.95, 1.21)	1.07 (0.96, 1.20)	1.07 (0.96, 1.21)	1.09 (0.97, 1.23)	0.415
ApoB (g/L)	0.79 (0.68, 0.93)	0.81 ± 0.18	0.83 (0.72, 0.95)	0.85 (0.75, 0.98)	<0.001
Current smoking (%)	205 (20.2%)	205 (19.3%)	228 (22.1%)	176 (17.1%)	0.280
Current drinking (%)	129 (12.6%)	147 (13.8%)	148 (14.3%)	128 (12.4%)	0.993

BMI: body mass index; SBP: systolic blood pressure; DBP: diastolic blood pressure; MAP: mean arterial pressure; AHI: apnea-hypopnea index; FBG: fast blood glucose; FSI: fast serum insulin; HOMA-IR: homeostasis model assessment of insulin resistance; TC: total cholesterol; TG: total triglyceride; LDL-C: low-density lipoprotein cholesterol; HDL-C: high-density lipoprotein cholesterol. Insulin resistance: HOMA-IR > 3.0.

## Data Availability

The data that support the findings of this study are available from the Department of Otolaryngology, Therapy Center for Obstructive Sleep Apnea, Shanghai Jiao Tong University Affiliated Sixth People's Hospital. But restrictions apply to the availability of these data, which were used under license for the current study, and so they are not publicly available. Data are however available from the authors upon reasonable request and with permission of the Department of Otolaryngology, Therapy Center for Obstructive Sleep Apnea, Shanghai Jiao Tong University Affiliated Sixth People's Hospital.
